# Models of the Translation Process and the Free Energy Principle

**DOI:** 10.3390/e25060928

**Published:** 2023-06-12

**Authors:** Michael Carl

**Affiliations:** Department of Modern and Classical Language Studies, Kent State University, Kent, OH 44240, USA; mcarl6@kent.edu

**Keywords:** free energy principle, active inference, translation process research, monitor model

## Abstract

Translation process research (TPR) has generated a large number of models that aim at explaining human translation processes. In this paper, I suggest an extension of the monitor model to incorporate aspects of relevance theory (RT) and to adopt the free energy principle (FEP) as a generative model to elucidate translational behaviour. The FEP—and its corollary, active inference—provide a general, mathematical framework to explain how organisms resist entropic erosion so as to remain within their phenotypic bounds. It posits that organisms reduce the gap between their expectations and observations by minimising a quantity called *free energy*. I map these concepts on the translation process and exemplify them with behavioural data. The analysis is based on the notion of translation units (TUs) which exhibit observable traces of the translator’s epistemic and pragmatic engagement with their translation environment, (i.e., the text) that can be measured in terms of translation effort and effects. Sequences of TUs cluster into translation states (steady state, orientation, and hesitation). Drawing on active inference, sequences of translation states combine into translation policies that reduce expected free energy. I show how the notion of free energy is compatible with the concept of *relevance*, as developed in RT, and how essential concepts of the monitor model and RT can be formalised as deep temporal generative models that can be interpreted under a representationalist view, but also support a non-representationalist account.

## 1. Introduction

Since the 1980s, empirical translation process research (TPR) investigates human translation and post-editing processes (e.g.,  [1,2,3]). While earlier research used think aloud protocols and retrospective interviews, since the mid-1990s, software tools (e.g., ScriptLog [4], InputLog [5], and Translog-II [6]) have been developed to record keystrokes in time. Ten years later, around 2005, eye-tracking technology was available that could be used to collect reading patterns on the source text (ST) and the evolving target text (TT). Thus, a complete log of behavioural patterns could be created which made it possible to investigate the relation between the input (gazing patterns) and output (typing behaviour) of translators.

In parallel with these technological developments, which resulted in numerous empirical investigations, TPR has also generated several cognitive models to explain the behavioural observations (e.g., [7,8]). Conceptualising and assessing translation *effort* has been at the centre of many TPR models. For example, Krings [9] (p. 178)) proposed that effort can be measured on three levels: temporal, technical, and cognitive. While technical and temporal effort can be measured with simple technical devices, operationalising cognitive effort has been considered more difficult, and defined in different ways, as “the amount of the available processing capacity of the limited-capacity central processor utilized in performing an information-processing task” [10], as the extent to which limited cognitive resources are exhausted [9] or an experience resulting from behaviour that diverges from preferences or habits [11].

One explanatory factor for cognitive effort has been the assumption of—and the distinction between—automatic and non-automatic processing, (e.g., [7,12,13]). Englund-Dimitrova [14] (p. 26), for instance, notes that “there are segments which are translated apparently automatically, without any problems [i.e., low effort], and other segments where the translation is slow, full of many variants and deliberations, which necessitates a problem solving approach and the application of strategies.”

These studies show that the translation process unfolds in terms of perception–action loops [15,16], which have been referred to as translation units (TUs). TUs constitute the empirical basis for some of the modelling in TPR (e.g., [17,18,19,20], as can also be seen in Section 2). TUs are thought to be the basic unit by which translations are produced. Malmkjaer [21], for instance, defines process-oriented TUs as a “stretch of the source text that the translator keeps in mind at any one time, in order to produce translation equivalents in the text he or she is creating” (p. 286). While Malmjkaer thinks of TUs as mental constructs, in this article (cf. Section 2), TUs are considered a combination of both mental events and physical (observable) behavioural acts.

Translation *effort* has thus been investigated in TPR in different ways based on behavioural traces captured during the translation process, whereas the most obvious translation *effect* is perhaps the final translation product. However, effects are also visible during the process, for instance, in the translator’s decision when selecting the most relevant path of affordances (i.e. a translation policy, as discussed in Section 6) amongst a large number of possibilities. Relevance theory (RT, [22,23,24,25]) provides tools to decide which of the many possible affordances should be taken. RT suggests a principle of relevance according to which human cognition is geared towards relevance. Relevance is a function of effort and effect: greater effect implies greater relevance while greater effort reduces relevance. RT formulates a relevance-theoretic comprehension procedure according to which humans aim to expend the least effort while aiming at maximum effects and they stop when the expectations of relevance are satisfied.

Besides RT, a number of models have been suggested to explain the process of translation (e.g., [1,2]). The monitor model (see [12], and also Section 3) stipulates that automatic priming processes are the basis of human translation production. However, the monitor model also assumes that translators may use, in addition, a set of higher-order, consciously accessible strategies which provide them with criteria to decide whether the produced translations actually correspond to the higher-order translation goals, aims, or guidelines (Box 1). While the automatised priming processes are quick and associated with low levels of effort, monitoring processes may require a substantial amount of translation effort. For instance, monitoring processes seem to disintegrate loops of concurrent perception–action into successive reading and typing activities (cf. [26]).

Box 1 **Priming and Monitoring in Translation.**

**Figure 1:**
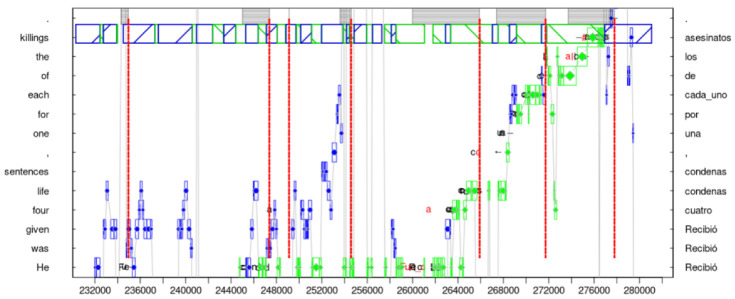
Sequence of TUs indicating a strategy to overcome a translation hurdle: the first TUs indicate hesitation and searching, followed by a short orientation phase, ending in smooth translation production.

According to *The decision Lab* “Priming, or, the Priming Effect, occurs when an individual’s exposure to a certain stimulus influences their or her response to a subsequent stimulus, without any awareness of the connection”, https://thedecisionlab.com/biases/priming (accessed on 7 March 2023). In this article, ‘priming’ is used in a broad sense, as an exposure to stimuli that (subconsciously) train our memory and lead to implicit bias. In the context of FEP and active inference, in Section 4, Section 5 and Section 6, this implicit bias can be formalised in terms of priors in a Bayes reasoning framework. Figure 1 provides an example where assumed priming effects lead to an incomplete, or mis-translation. The figure plots a problematic translation from English “*four life sentence*” into Spanish “*cuatro condenas*” (i.e., missing the translation of “life”) which suggests the dominance of automatised, stimulus-guided processing routines and a lack of (successive) monitoring/higher-order cognitive control. RT may explain this gap in terms of relevance, i.e., as an unbalanced trade-off between worthwhile effort and effects. Following a stretch of extended hesitation and revision in an earlier part of this translation (see Section 2), a continued reflection or revision of this part of the translation may have been—for the translator—too effortful and thus of insufficient relevance at this point. See Section 2 for further discussion.

This article proposes to model the translating mind within the framework of the FEP [27,28] and active inference [29]. The FEP provides a general theory (a principle) as to how living organisms maintain their existence by minimising entropy and surprise. FEP posits that living systems must reduce the discrepancy between their expectations and observations, which can be measured as an information-theoretic quantity, referred to as *free energy*. Active inference builds on the FEP and introduces the notion of *expected free energy* which relates to planning and action policy selection. The article argues that FEP and active inference have the potential to integrate central claims of RT (as derived from the principle of relevance) and of the monitor model (i.e., different strata of slow and fast processing) into one consistent and mathematically sound framework.

Section 2 illustrates examples of translation processes that exhibit different levels of effort and effect, where the translation process is fragmented in terms of loops of (ST) reading and (TT) typing. TUs may thereby exhibit properties of automatised and/or monitoring processes. Section 3 provides a brief overview over the monitor model and an extension that accommodates assumptions of RT. Section 4 introduces the basic ideas of the FEP while Section 5 discusses active inference [29] and addresses how action policies are selected. It discusses an operationalisation of priming, effort, and relevance in the context of active inference. Successively, Section 6 relates the FEP to translation process data and discusses examples of behavioural data that show how translators tune themselves in a flow of translation production. It argues that the principle of relevance can be cast in terms of the free energy. Section 7 argues that—within the framework of active inference—the translation process can be understood in representationalist and in non-representationalist terms.

## 2. Translation Units

Translation units (TUs) are empirical constructs that segment the flow of translation activity into successive units which “reflect entities of coherent cognitive activities” [18]. Drawing on ecological-enactive theory [30,31]), Carl [32] conceptualises TUs as *translation affordances*: affordances, Baggs and Chemero [33] say, are relations between the features of the environment (i.e., the ST) and the abilities of an agent (e.g., translation skills); they are “opportunities for behavior, which [...] are the main things that animals perceive” ([33] p. 3). According to Chemero [31] (p. 152), environmental features and abilities “causally interact in real time and are causally dependent on each other”. They are perceptually re-assessed at each moment so that a different factorisation may result in different affordance probabilities over time. This is also what we observe in the translation process. Franchak and Adolph [34] add that affordances could be modelled in terms of probabilities: “affordances are better considered as continuous, probabilistic functions that represent an individual’s likelihood of successful performance” [34] (p. 2).

Rietveld and Kiverstein [35] introduced the notion of the *field of affordances*: a field of affordances amounts to the (often exponentially large) number of possibilities for action that an agent could engage in at any moment in time. However, there are only a few relevant affordances—so-called *solicitations*—that agents actually realise. While there is, theoretically, an exponential number of ways in which a text (or sentence) could be split into pieces, in practice, the number of observed TUs are quite limited. The sequence of selected and realised *solicitations* that make up the path of observed TUs is likely to be much smaller and will show typical probabilistic distributions, which depend on a number of parameters, such as expertise, text difficulty, translation guidelines, etc [16,19,36]. Translators usually follow the sequential order of the ST, translating the ST piece-by-piece, thereby reproducing approximately the structure of the source in the target.

With a view from active inference, a further distinction between *epistemic affordances* and *pragmatic affordances* has been suggested [37]. Epistemic affordances are exploratory—an agent scrutinises the environment to resolve uncertainty—while pragmatic affordances exploit previously allocated resources. I return to this distinction in Section 5.

Figure 1 provides an example that shows how the translation process can be fragmented into sequences of TUs. It shows a progression graph in which a translator translates the sentence *’He was given four life sentences, one for each of the killings.’* into Spanish. The left vertical axis in Figure 1 plots the source text, from bottom to top, that is being translated. The right vertical axis shows the Spanish equivalents of the words and expressions on the left. The horizontal axis represents the time in ms in which the Spanish translation was produced (approximately 50 s). The graph shows gaze activities on the source (blue asterisks) and on the target text (green diamonds), as well as keystrokes (black) and deletions (red). The graph fragments the translation process into eight TUs, as shown by red double-lined demarcations. Each TU is defined by a production burst (i.e., sequences of uninterrupted typing) and a preceding typing pause. Production units are marked as striped blocs within each TU.

The sequence of TUs in Figure 1 suggests that the translator first struggles with the rendering of *‘He was given’* in the first 30 s or so (five TUs) in which she re-reads several times the first ST words, and types and deletes several solutions until finding a Spanish solution *Recibió*. Around time stamp 234,000, the translator first types a (preliminary) translation solution *‘Fue’* which is, however, later deleted. That is, the flow of production is interrupted and the translator re-reads the first few words a number of times until about 12 s later (around timestamp 246,000), an incomplete solution is generated (*‘condenad’*). It is likely that, during these 12 s, some amount of reflective thought, i.e., monitoring processing and internal searching, has taken place. Once a solution is found, the translation goes on smoothly with no delay, mainly monitoring (green diamonds) how the typed text appears in the target text editor (black: insertions, red: deletions).

**Figure 2:**
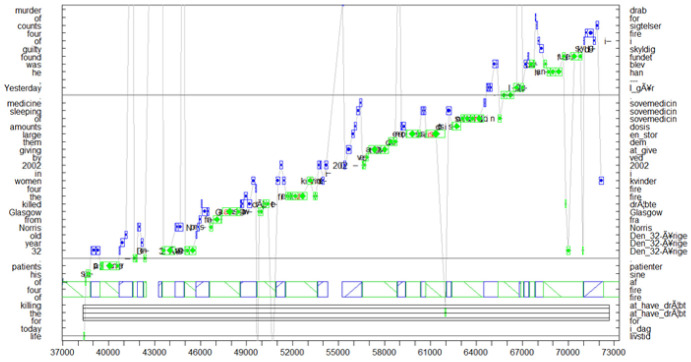
Translation unit of highly skilful translator–environment coupling with minimal look-ahead in the ST and immediate TT production.

I will argue, in Section 4, that the hesitations and re-formulations, as illustrated in the first three TUs in Figure 1, suggest that the translator is in a state of surprise, which can be defined in terms of *free energy* as a consequence of low (processing) *accuracy*. The hesitation and the related *processing inaccuracy* is further corroborated 14 s later (around timestamp 260,000) where the preliminary translation solution is deleted and replaced by Spanish *’Recibió’*. This translation option only seemed to have occurred to the translator after reading ahead in the ST (in TU${}_{4}$, roughly between timestamps 250,000 and 254,000), with the preview of a larger ST context. However, after this initial hurdle is overcome, the translator in Figure 1 then turns into a smooth, automatised translation flow similar to the situation shown in Figure 2 (however, see discussion in Box 1).

Figure 2 shows a progression graph in which an ST segment of 30 words is translated from English into Danish in approximately 35 s. The 30 words cover parts of three sentences (sentence boundaries marked as horizontal lines). As can be seen in the graph, in most cases, the translator’s eyes are approximately between two and four words ahead in the ST, for which a translation is currently typed. That is, translation proceeds monotonously, almost word-for-word over the entire segment, with an eye-key span of approximately four words. Only at one moment, around timestamp 55000, does the translator read a small phrase, around seven words ahead in the ST, however, without any noticeable (i.e., longer than 1 s) interruption in the typing flow. Such almost immediate eye–hand coordination suggest highly skilled translators, which are able to instantaneously translate and type out sequences of short (minimal) segments of input text. We assume that such ‘peak-performance’ [38] is the preferred, most productive translation state. Here, presumably automatised translation routines dominate the production process, as suggested by the apparently effortless but effective, incremental generation of partial translation solutions that seamlessly connect to the preceding and following chunks. Figure 2 exemplifies how translators can be in a steady flow of interaction with their environment in which policies of perception–action loops are in tune with preceding expectations and textual predictions.

In Section 4, Section 5 and Section 6, I will explain such sequences of smooth production in terms of minimising free energy. Free energy is a measure for how well the agent’s expectations coincide with the current sensory input plus how likely a perceived event is. It is a proxy for surprise, which reflects the (im)probability of finding an organism in some sensory state [39,40]. That is, high levels of production *accuracy* (Section 4, Equation (2)) and/or low amounts of *expected ambiguity* (Section 5, Equation (5)) may lead to a reduction in surprise in the successive reading and translation patterns that characterise the steady translation state.

How likely are those different translation behaviours? While there are a large number of parameters that determine behaviour (e.g., expertise, text difficulty, translation brief, workspace conditions, fatigue, hunger. affect, etc.), the investigation in [41] suggested that only slightly more than 3% of TUs in their experiment indicated some sort of ‘higher-order’ meta-representationalist activity, that is, macro units that show a revision of content words. Skilful translator–environment coupling—as depicted in the TU of Figure 2—seems to be rather common, where it seems that cognitive activities are limited to finding suitable local collocations.

## 3. The Monitor Model

**Figure 3:**
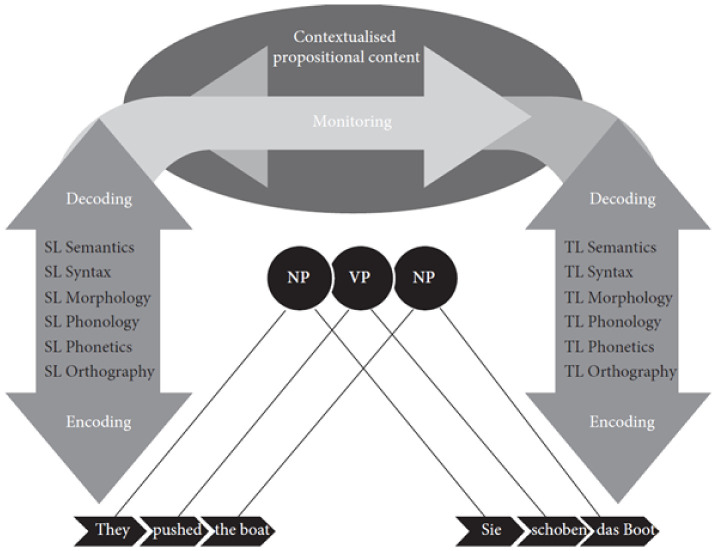
The monitor model, taken from [12].

While TPR findings have been explained with several theoretical models, the monitor model ([12], see Figure 3) is—to the best of my knowledge—the only theoretical framework within cognitive translation studies (CTS) which takes into account basic (priming) and higher-order (monitoring) processes in translation. It is inspired by the work of Tirkkonen-Condit [42] (p. 405), who posited the existence of a "translation automaton and its monitoring mechanism" to interact in human translation. The monitor model of Schaeffer and Carl [12] (p. 21) has been primarily designed to “investigate automated processing during translation” and to integrate automatic processes (basic cognition) with ‘higher-order’ controlled monitoring processes. It builds on De Groot [43], who described two different points of view that she calls vertical and horizontal translation. The horizontal processes build on research in psycholinguistics (e.g., [44]) and bilingualism (e.g., [45]), whilst vertical processes correspond to the distinction of linguistic strata, as shown in Figure 3. The monitor model assumes that the horizontal processes are based on the cross-lingual, non-selective activation of shared representations while the vertical processes are language-specific, higher-order constructs that require a comparison mechanism to verify the similarity of the two monolingual (SL and TL) analyses. In this account, the horizontal processes rely on quick priming mechanisms, while the vertical monitoring (and revision) processes take up the bulk of translation effort.

**Figure 4:**
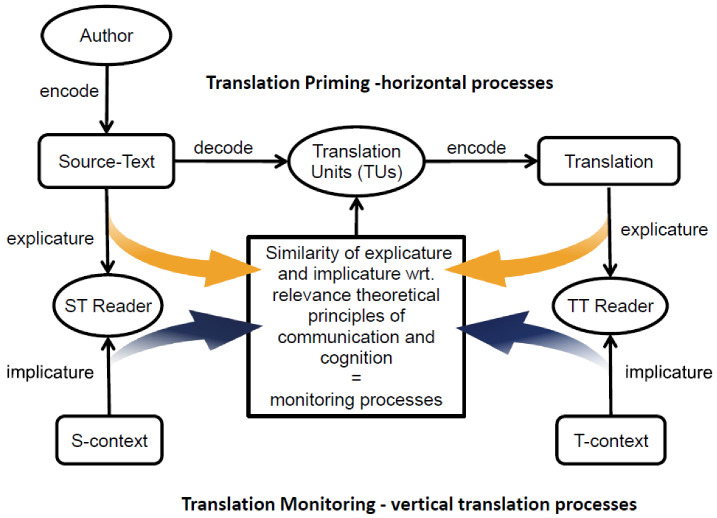
RT and the monitor model: horizontal processes fragment the ST into translation units, while vertical processes control for interpretive resemblance, based on explicitly and implicitly encoded information in the source and target (adapted from [46]).

Carl and Schaeffer [46] draw on RT [24,47] to address the un(der)specified monitoring function. They propose an extended noisy channel model that integrates translation priming (horizontal processes) with components of RT. As in the original monitor model, they assume that priming is an automatism by which translators retrieve and generate translations, while the monitor has access to additional cognitive resources to check the similarity of the source and the target. Figure 4 shows a slightly modified version of the [46] model, which stipulates that the noisy translation process is fragmented into translation units (TUs) and controlled by monitoring processes.

Gutt [24,47] defines translation as an activity of *interpretive language use* which establishes correlations of “bodies of thought” [47] (p. 13) across different languages, rather than similarities of propositional truth between expressions of source and the target texts. In his later writings, Gutt [25] makes a distinction between two modes of interpretive language use (i.e., translation). The stimulus mode (s-mode) informs the audience of “what was said” i.e., focusing on *explicatures*, while the interpretation mode (i-mode) informs the audience “what was meant”, taking into account *implicatures* (see Figure 4). In this model, translation can be mainly based on the ‘s-mode’ when there are large commonalities in the communicative environment of the source and the target audience. Otherwise, the i-mode may help translators bridge communication barriers. In this view, the s-mode is largely based on priming processes, while monitoring processes can be expected to become involved if translations are to be produced in the i-mode. For example, Carl and Schaeffer [46] (see Figure 4) suggest modelling vertical monitoring processes to complement the noisy channel “by adding constraints of causal interrelation between stimulus, context and interpretation, established by the principle of relevance” [46] (p. 60).

The principle of relevance [23,48] is a central concept in RT which stipulates that human cognition aims to maximise relevance. RT defines *relevance* as the trade-off between *effort* and *effect*: greater effect implies greater relevance while greater effort reduces relevance. It also posits that human communication follows a relevance-theoretic comprehension procedure [23] (p. 30):

1. Follow a path of least effort in computing maximum cognitive effects;
2. Stop when your expectations of relevance are satisfied.

In the translation context, it can be assumed that both, the i-mode and s-mode, are driven by the principle of relevance, depending (among other things) on the (assumed) similarities of the cognitive environment of the source and the target audience, the translation purpose (i.e., as communication by means of translation guidelines), translator expertise, etc. According to RT, translators strive to optimise the relevance of translations by maintaining the interpretive resemblance of implicatures and explicatures (i.e., maximising the translational effect) across potentially different cognitive environments associated with the source and target texts while at the same time expending the least cognitive effort.

Wilson and Sperber [23] reject the possibility of quantifying effort, effect or relevance: “Since we are interested in relevance as a psychological property, we have no reason to aim for a quantitative definition of relevance” (Sperber and Wilson [48] (p. 132)). They argue that effort and effect are non-representationalist dimensions of mental processes: “relevance is a property which need not be represented … [but] when it is represented, it is represented in terms of comparative judgements and gross absolute judgements, (e.g., ’irrelevant’, ’weakly relevant’, ’very relevant’), but not in terms of fine absolute judgements, i.e., quantitative ones.” [48] (p. 132).

In this article, we deviate from these assumptions and suggest that the FEP/active inference is a mathematical framework which is suitable for quantifying and predicting mental processes in translation.

## 4. The Free Energy Principle (FEP)

The FEP suggests that living beings are open systems which are in constant exchange with their environment [27]. While the environment produces sensory impressions that the agent can adapt to, the organisms act on the environment and—as a consequence—change the states in the environment, which in turn changes the observations. The FEP and active inference claims these to be characteristics for any self-organising system which applies equally to biological systems, from single-cell organisms to complex animals, as well as to cognitive systems and social networks [29] (chapter 10). It is a powerful theory which relies on the assumption “that any adaptive change in the brain will minimize free-energy.” [27] (p. 293) Within these dynamics, living organisms maintain their existence by minimising the information-theoretic quantity of variational free-energy.

**Figure 5:**
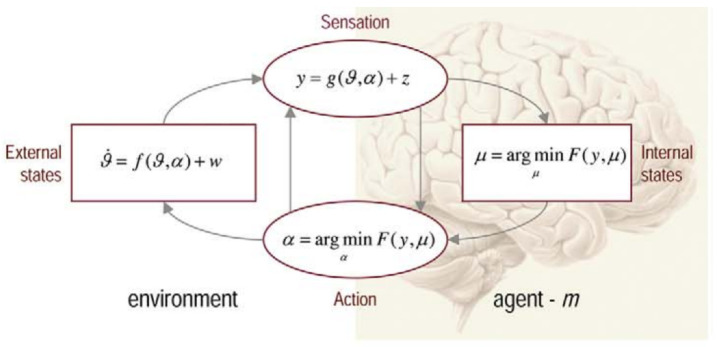
The free-energy principle [27] consists of brain states: μ, sensory input: y, action: α, environment: ϑ.

Figure 5 plots an instantiation of the FEP and its parameters. It shows the relation between internal brain states μ, actions α, sensory input y, and external (or environmental) states ϑ. Figure 5 shows the circular relation between the (internal) brain states and the (external) environmental states which are separated through sensation (y) and action (α), so-called Markov blankets (MBs) [49,50,51]. Sensation, in this view, is a bottom–up process to receive outside stimuli. Perception is a top–down process considered to organise and contextualise the sensory information and serves as an input for action. From the point of view of the organism—i.e., a view from the internal brain states—the external world is ’hidden’ and can only be accessed through sensation (y) and (indirectly) through action (α), i.e., to the extent that action changes the way that the environment is sampled.

States with a high amount of uncertainty (high entropy) possess a large amount of (variational) free energy which reflects a ‘disattunement’ [51] between the agent and environment, for instance, a gap between expectation and the sensory input. The brain can minimise this free energy either through sensory input and observation, i.e., when inferring the cause of sensory input (y) and accordingly adjusting the internal model (μ) to better fit the observation, or through actions (α), by which the subsequent sensory input becomes more similar to what is predicted by the model. Either of the two optimisation pathways leads to a minimising free energy which warrants the survival of the agent and/or a smooth agent-environment interaction: “[b]y minimizing free-energy, the organism resists entropic dissipation and maintains itself in its phenotypical steady-state.” [51] (p. 72). Friston says: “Agents can suppress free energy by changing the two things it depends on: they can change sensory input by acting on the world or they can change their recognition density by changing their internal states. This distinction maps nicely onto action and perception” [28] (p. 129). I will take up this point in Section 5.

The FEP assumes that the brain uses a generative model—e.g., a Bayes network—that can be used for the selection and analysis of relevant affordances [37]. In this view, free-energy is a proxy for the self-information (i.e., the surprisal) of a state which can be reduced by changing brain states (μ) and/or action (α). The notion of blanket states is central in this respect: internal states (e.g., states in the brain) and external states are conditionally independent and can only influence one another through blanket states. In Figure 5, sensation (y) and action (α) form MBs which are sets of states that statistically isolate internal states (μ) from external (or hidden) states (ϑ).

(1) $$\begin{array}{cc} F_{\mu } & =\sum_{\mu }q\left(\mu \right)\log_{2}\left(\frac{q(\mu )}{p(y(\alpha ,\vartheta ),\mu )}\right) \end{array}$$

Friston [27,28] formalises free energy ($F_{\mu }$), as shown in Equation (1), whereas q(μ) are the densities over expectations as maintained by the internal model, and p(y(·)|μ) are the probability densities of observations given the model. If both distributions are identical (the observations fit the expectations), the term equals 1. Accordingly, its log value becomes zero, and so does the free energy. The more expectations (q(μ)) diverge from observations, the higher the free energy will be.

According to Wei [52], Levy [53] makes out two reasons for disruption in real-time sentence processing. On the one hand, *resource allocation* relates to memory processes, such as the lack of cognitive resources for the storage and retrieval of units necessary to analyse the linguistic input. On the other hand, unexpected input—i.e., sensory surprise—causes a shift in resources, *resource relocation*, e.g., by acting on the world. Two sets of theories are related to investigating this differential processing difficulty: The resource allocation approach is, according to Wei, the dominant approach in psycho-linguistic research, concerned with memory processes, while resource relocation theories are concerned with the change in the conditional probability distribution over all interpretations [53].

(2) $$\begin{array}{cc} F_{\mu } & =\overset{\mathrm{complexity}}{\overset{︷}{D_{KL}\left(q\left(\mu \right)\|p\left(\mu \right)\right)}}-\overset{\mathrm{accuracy}}{\overset{︷}{\sum_{\mu }q\left(\mu \right)\log_{2}p\left(y\left(\alpha ,\vartheta \right)|\mu \right)}} \end{array}$$

The FEP addresses both phenomena. The factorisation in Equation (2) splits the free-energy $F_{\mu }$ into two terms, namely *complexity* and *accuracy*, which account for resource allocation and resource relocation, respectively (see, e.g., Solopchuk tutorial: https://medium.com/@solopchuk/tutorial-on-active-inference-30edcf50f5dc, accessed on 6 March 2023). The Kullback–Leibler divergence ($D_{KL}$, on the left in equation (2)) quantifies the difference (i.e., the approximation) between the expected states q(μ) and observed states p(μ). This term is referred to as *complexity* within the FEP framework as it indicates the number of bits needed for encoding extra information in *q* to account for the observation *p*. If both distributions are identical, no extra bits are needed, and thus no additional resource allocation is required, which implies that complexity is minimal.

The term on the right indexes the *accuracy* of (translational) action. The success of resource relocation can be measured in terms of the surprise that an action causes on a forthcoming observation. It is the surprise of a (successive) joint observation α and ϑ given a distribution of states μ. Resource relocation consists of adjusting the environment through action (α) so as to minimise the surprise of successive sensory input (y(α,ϑ)). Thus, the increased accuracy of (e.g., translational) action leads to higher coherence with the successive expectations (q(μ)) and thus lowers the average surprise.

There may thus be a significant correlation between the two notions of production and translation accuracy (fluent production vs. free of translation errors). The notion of accuracy refers, herein, to the smooth flow of the production process rather than the amount of (translation) errors produced in the outcome of the task, as this term would be used in translation quality assessment. However, previous research indicates that sequences of smooth production are also less prone to translation errors than stretches of hesitation and revision (cf. Carl and Báez [54], despite the apparent counter-example in Box 1). Note that the accuracy term in Equation (2) will be ≤0 and the most accurate action amounts to 0, since it contains (the sum of) $\log_{2}$ probabilities.

As discussed, e.g., in (Parr et al. [29], Smith et al. [49]), free energy can be factorised in different ways. In Equation (2), it is the “trade-off between accuracy and complexity” [55]. Minimising free energy corresponds to minimising complexity while maximising the production accuracy. As a corollary, FEP predicts that organisms sample the sensory information that conforms to their expectations, and that the best internal model describes the data in the simplest and most accurate manner.

## 5. Affordances, Action Policies, and Active Inference

Ecological-enactivist approaches to cognition (e.g., [30,31,37,51]) relate action and perception to the notion of *affordances*. The term has been used to suggest that perception can be a direct guide to action, without a need for higher-order mental representation [30,31]. For Gibson [30], affordances are configurations of environmental features that allow/invite for action. Behavioural flexibility allows for different ways in which affordances are learned, detected, and selected. Gibson and Pick [56] maintains that affordances can be learned through a process of differentiation by splitting a more general affordance into multiple, more specific affordances and associated actions. Similarly, Gallagher [57] (p. 200) posits that “[o]ur ability for making sense of the world comes in part from an active and pragmatic engagement with the world” where the world is laid out “in terms of differentiations that concern my action possibilities”. Rather than re-constructing or re-presenting the world, this view suggests that the brain is an organ capable of enabling representationally unmediated action that can flexibly attune to changing circumstances.

Affordances are typical for the type of niche in which an agent lives. Ramstead [51] (p. 167–168) makes a distinction between two types of niche construction: On the one hand, selective niche construction (SNC) refers to phylogenetic (evolutionary) changes in the environment—SNC, he says, is a way for organisms to selectively interact and construct their environment (i.e., the *umwelt*) can have potential evolutionary significance; On the other hand, developmental niche construction (DNC) operates on the scale of learning (ontogenetic development) and perception–action cycles. While niche construction often refers to ecological niches and evolutionary biology, human culturally transmitted and learned niches—including language use—are (at least) equally important types of niche construction. Ramstead [51] (p. 167) points out the intrinsic nature of interaction between social and cognitive aspects of affordances, in which collective behaviour provides affordances—that is, sets of possibilities for engagement with the niche—”that become relevant from the point of view of the needs and concerns of any single agent”.

Subsequently, for Chemero [31] (p. 60), “affordances are relations between an animal’s abilities to act and a situation in the world”. In order for targeted action to happen, those action opportunities must be detected and realised, so that in the context of “active inference, [an] affordance becomes an attribute of the plan or course of action” (Friston [37] (p. 2017)). In this view, the breaking down (splitting) and differentiation of environmental configurations into sequences of specific affordances and their associated actions can be cast as action policies (π). Action policies “show how organisms select the actions with the most adaptive value, which intrinsically lends itself to a pragmatist or enactivist reading.” (Ramstead [51] (p. 53)) Active inference thereby determines the selection of possible action policies: “active inference is a theory of action policy selection” [51] (p. 53). It is “a corollary or process theory that follows from the free energy principle” (Friston [37] (p. 211)).

Depending on whether affordances minimise prediction errors or expected surprise, they have been distinguished as having epistemic or pragmatic ‘values’: epistemic affordances resolve uncertainty through ‘epistemic foraging’ while pragmatic affordances “ensure outcomes that I find unsurprising and comfortingly familiar” (Friston [37] (p. 216)). In this view, affordances “can be selected from plans that minimise prediction error or surprise expected after acting” [37] (p. 215). Similarly, Gallagher [57] (p. 18) sees two directions of fit in this process:

[t]he first involves updating predictions or updating priors on the basis of ongoing perceptual experience–the world-to-brain direction. The second involves acting on the world to directly shape or re-sample it in such a way as to directly test our prior expectations… for example, active ballistic saccades do not merely passively orient towards features but actively sample the bits of the world that fit my expectations or resolve uncertainty.

Under these definitions, active inference is the process of organisms planning their actions on the ecological niche in the world and updating their internal states accordingly. If we change the environment, then our sensory input also changes, where the best sequence of action most efficiently makes the world more like our predictions. "Therefore, action can reduce free-energy (i.e., prediction errors) by changing sensory input, whereas perception reduces free-energy by changing predictions" (Friston [27] (p. 295)). Active inference enables active behaviour which allows organisms to maintain states of viable, adaptive coupling within their ecological niche. In this view, paths through fields of affordances can be quantified and evaluated by the expected free energy gradients of the selected action policy.

More formally, an action policy π is a sequence of actions and active inference is the joint optimisation of a sequence of actions; thus, action policies (π) are selected that minimise expected free energy.

Equation (3) shows how action policies (π) may be taken into account in this framework [49]. Equation (3) is similar to Equation (1), but probabilities are now conditioned on action policies π. Action policies determine preferred sequences of action that are likely to minimise the expected free energy $G_{\pi }$ in the best possible way. Equation (4) introduces the term $\sum_{\mu }q\left(y\left(\alpha ,\vartheta \right)|\mu \right)$ as a weighted sum over the prediction errors ($\log_{2}\left(\frac{q(\mu |\pi )}{p(y(\alpha ,\vartheta ),\mu |\pi )}\right)$). This amounts to the fact that future actions and observations follow informed probability distributions.

Equation (4) can be further split into two factors [29,49], (as can also be seen in Solopchuk tutorial), namely the *expected risk* and the *expected ambiguity*, as shown in Equation (5). The expected risk is the divergence between (the probability of) the expected observations under the policy π and the (probability of the) prior observation. The expected ambiguity measures the uncertainty (entropy) of possible actions and their presumed successive perception H(p(y(α,ϑ)|μ), weighted by expectations under the policy π. Minimising these factors results in the selection of a preferred policy.

(3) $$\begin{array}{cc} G_{\pi } & =\sum_{\mu }q\left(\mu |\pi \right)\log_{2}\left(\frac{q(\mu |\pi )}{p(y(\alpha ,\vartheta ),\mu |\pi ))}\right) \end{array}$$

(4) $$\begin{array}{cc} \; & =\sum_{\mu }q\left(\mu |\pi \right)\sum_{\mu }q\left(y\left(\alpha ,\vartheta \right)|\mu \right)\;\log_{2}\left(\frac{q(\mu |\pi )}{p(y(\alpha ,\vartheta ),\mu |\pi ))}\right) \end{array}$$

(5) $$\begin{array}{cc} \; & =\overset{\mathrm{Expected Risk}}{\overset{︷}{D_{KL}\left(q\left(y\left(\alpha ,\vartheta \right)|\pi \right)\|p\left(y\left(\alpha ,\vartheta \right)\right)\right)}}-\overset{\mathrm{Expected Ambiguity}}{\overset{︷}{\sum_{\mu }q\left(\mu |\pi \right)H(p\left(y\left(\alpha ,\vartheta \right)|\mu \right)}} \end{array}$$

The framework allows the notions of effort and relevance to be operationalised. According to Parr et al. [11], cognitive effort can be defined as the divergence between a context-sensitive belief about how to act, and a context-insensitive prior belief such as habits or preferences. It is “the qualitative experience of committing to a behaviour that diverges from a priori habit” [11] (p. 2). Thus, in this view, outcomes are least effortful when acting according to prior preferences and/or habits. Presuming effects are equal, a policy that realises prior beliefs is then also the most relevant for the agent.

Under these considerations, priming can be understood as a process of generating preferences (or prior beliefs) followed by a policy that enacts a sequence of context-insensitive responses which are (implicitly) biased towards these prior beliefs. That is, in a primed task, a first stimulus (the prime) activates a set of priors and successive stimuli trigger automatised responses that are biased toward these prior preferences, without (immediate) awareness of their connection(s). Answering to a stimulus without intervening (reflective) thought is easy and fast, which requires low levels of cognitive effort and is, thus, the most relevant policy in situations that presume low levels of expected risk and/or expected ambiguity. It is also associated with the lowest levels of free energy.

Note that the outputs of actions are inputs to successive observations, so that an agent may enter a cycle of ‘self-priming’ or self-evidencing—where a cycle of integrative (self-) priming [58] is a form of self-evidencing. Accordingly, Kirchhoff and Kiverstein [59] (p. 4901) posit that a “system is self-evidencing because it is a free-energy minimising system.” Any system, they say, that minimises prediction errors will be, in the long run, self-evidencing, where self-evidencing “is equivalent to the agent maximising evidence for the hypothesis of its own continued existence in its niche.” [59] (p. 4901).

It can thus be assumed that the amount of effort spent on a task is tractable in action and sensation (sensory input). Sensory input, on the one hand, has the potential to trigger changes in internal states. Extended sequences of visual search may indicate extended effort due to a need (or wish) to update the internal model, adjust posterior densities, or generate policies that reduce expected risk and ambiguity.

Actions, on the other hand, lead to effects that impact (future) sensory inputs. The surprise of a (future) sensory input depends on the accuracy of the action(s) and their connectivity with the external environment. Actions in the configurations of high (expected) ambiguity may increase successive confusion and hesitation. However, actions may also resolve environmental ambiguities such that they effectively lead to successive stable, self-generated sensory states. In this second case, an agent seeks to generate actions which produce observations that reduce uncertainty about external states.

## 6. Discussion

This section provides a sketch for a framework to conceptualise the translation process from an FEP/active inference point of view: the ST corresponds to the hidden (i.e., external) states (ϑ) where its constituents—i.e., the ST words, phrases and sentences, discourse structure, etc.—follow a probabilistic distribution. The translator’s internal states (μ) and the external states (ϑ) are separated by an MB. Sensory input comprises reading patterns, y, (i.e., gaze patterns) and actions are realised as typing activities, α, (i.e., translation production). Logged traces of the reading and writing patterns can be observed at the MB, and the recorded records of these behavioural data make up the translation process data (TPD) that form the basis of our analysis.

**Figure 6:**
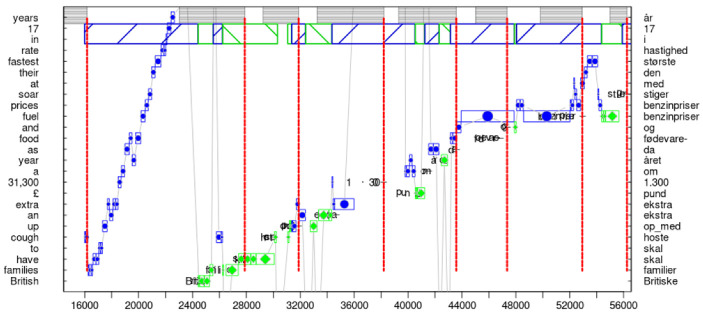
Translation policy of a large context planner: initial reading of the segment minimises the *complexity*, followed by highly *accurate*, apparently automatised production routines that, in turn, lower the surprise of successive reading patterns.

Parr et al. [29] (p. 46) point out that an agent must specify what optimal behaviour consists of, and a model must “specify the preferred conditions for the agent’s existence, or the regions of states that the agent has to visit to maintain its existence, or satisfy the criteria for its existence in terms of occupying characteristic states.” In that respect, RT suggests that “optimal behaviour” can be expressed in terms of relevance, i.e., as the trade-off between effort and effect. As discussed in Section 2, the trade-off between effort and effect in the translation process is enacted in loops of perception–action (i.e., affordances), conceptualised as TUs as, for instance, depicted in translation progression graphs in Figure 1, Figure 2 and Figure 6.

Section 4 and Section 5 argue that the notion of free energy is suited to (re)formulate relevance in quantitative terms. Free energy is the discrepancy between expectations and observations that can be reduced by adjusting the internal model or by changing the world. As suggested in Section 2, behavioural traces of the translation process elicit *effort* which can be assessed via, e.g., fixation duration, regressions and re-reading, deletions and revisions, pausing, internal or external search, etc. Indicators of high translation *effect*, on the other hand, include smooth and fluent typing, short and minimal look-ahead into the ST or TT before typing, the monitoring of TT production, etc. The relevance of a TU can then be assessed as the ratio of effect and effort indicators, where higher effects and/or lower effort lead to greater relevance. This definition is consistent with [11], as discussed in Section 5.

As by definition, every TU contains at least one typing event (a keystroke) preceded by a typing pause of at least one second [17,18]—and many TUs will also comprise gaze data—a relevance indicator can be determined for every TU based on the distribution of behavioural (process) and textual (product) data it contains. Furthermore, as each TU starts out with a typing pause (higher effort) and ends in a typing burst (higher effect), the free energy in the beginning of TUs will be (on average) higher than at the end, so that we will be able to determine a relevance gradient of free energy for every TU.

In line with the principle of relevance, translators aim to increase the relevance of their translational action. Thus, translators aim to reach a state of smooth and fluent production and they seek to minimise instances of hesitation or confusion. This coincides with the assumptions of active inference which predicts that agents seek to lower free energy: instances of hesitation or confusion indicate a high amount of free energy, whereas during sequences of fluent translation production, the energy is bound in the unfolding translation process. Translation relevance and free energy should thus be in a reverse proportional relation, i.e., a higher amount of free energy indicates lower relevance, and vice versa. On the basis of these considerations, we can categorise sequences of TUs into three internal states μ::{S,O,H}:

S A steady state results in a fluent translation production, with minimal look-ahead followed by immediate translation production. The steady states are characterised by high production accuracy—in a sense that produced chunks of translation do not result in hesitation in successive TUs. Following Friston [37], a steady state may be considered a kind of *pragmatic* affordance, which is associated with high levels of effects and low levels of free energy. An instance of the steady translation state is shown in Figure 2, and discussed in Section 2.
O A state of translation orientation can be observed as an extended ST reading patterns in which a translator explores the co-text (or other resources). An orientation phase is likely to adjust expectations based on the empirically gathered evidence (e.g., through ST reading) and thus result in more precise priors for successive production. A state of orientation may be considered an *epistemic* affordance, which is associated with higher levels of free energy.
H A state of hesitation or confusion shows either a “lack of determination (not knowing exactly what comes next out of several options) or overdetermination (current activity continues pre-reflectively while we are reflectively aware that it is no longer the relevant activity)” (Di Paolo et al. [60] (p. 4)). This translation state may be the most complex and difficult to describe and to operationalise, as there can be a large number of different reasons which may result in different behavioural patterns.

Whilst it is possible to assume additional internal states, such as, e.g., “Revision”, in this paper, I consider only these three states. Translation policies π are then sequences of translation states—which may consist of partial or several TUs—that translators pursue to accomplish their translation jobs. A policy is thereby a strategy in which the steady state (S) would be the preferred state that a translator aims to arrive at and to maintain, since it has—on average—the lowest levels of free energy. Translation policies π can be expressed as a regular expression of the form shown in Equation (6).

(6) π::(H|O)+S

In this context, we can re-consider the notion of translation affordances. Section 2 identified TUs as translation affordances, but the notions of translation states and translation policies give us a broader view of the translation process. In this view, a translation policy is a more general affordance that splits into multiple, more specific affordances and associated actions, e.g., a sequence of TUs. According to Friston [37] (p. 217), affordance, sensation, and action are closely interwoven, an “affordance becomes an attribute of the plan or course of action” such that “one cannot separate affordance—as an attribute of the thing to be acted upon–from the acting per se”. He suggests that epistemic affordances (i.e., translation orientation) and pragmatic affordances (i.e., fluent translation production) are ‘attributes’ of one policy, which cannot be separated, as they are tied together as parts (i.e., ‘values’) of the same translation policy.

We might also be able to determine affordances on a still larger level, for instance, when a translator accepts a translation job, considering whether they have the time and ability to engage in the job. It thus appears that we can make out embedded levels of affordance, where each level may be characterised in terms of different features and values, with different notions of relevance, effort, and effect. Further research may be needed to determine those different notions of relevance and their correlations.

Figure 6 provides an example of a translation policy (OS), an affordance that consists of an orientation (O) followed by a steady state (S) of fluent production. It illustrates the tight connection between *epistemic* and *pragmatic* affordances, as well as the splitting of a translation policy into successive TUs.

Figure 6 shows a 40 s sequence of behavioural data, fragmented into seven TUs, in which a Danish translation for 27 English ST words is produced. The progression graph shows two phases which are characteristic of a *large context planner* [61]. In the first six seconds, the translator engages in an epistemic part of the translation affordance by carefully reading (exploring) the source segment. Gaining a basic understanding of the sentence will likely allocate (prime) the appropriate mental resources that are useful in the successive pragmatic part of the affordance. For instance, it gives the translator the possibility to check whether they face any terminology or translation-related problems. In this case, the translator would probably consult external resources (lexicon, collocation tools, web, etc.) to search for missing translation knowledge and clarify any open translation hurdles. This can also be achieved through the consultation of internal resources. For instance, around time stamp 200,000 in Figure 6, a regression can be observed for *a year* which is part of the object for an unusual metaphorical expression (*cough up an extra £ 31,300*). It is possible that there is a relation between the regression and unusual metaphor. However, this metaphor did not seem to raise further problems for this translator, perhaps because the fragment was literally translated into Danish (*hoste op med*).

The monitor model [12] suggests that an orientation phase might activate (subliminal) translation routines and translation patterns to be readily available in the successive translation drafting phase. In line with the assumptions of active inference, an orientation phase may thus result in adjusting expectations by closing gaps between the internal states and observations of the external ST, lowering the expected risk and ambiguity of the model. This enables the translator to efficiently engage in the pragmatic part of the affordance.

The successive TUs in Figure 6 seem to corroborate this assumption. Long stretches of fluent typing patterns and the tight coordination of reading and typing behaviour indicate a translation policy associated with a low amount of risk and ambiguity (cf. Equation (5)). In the first 10 seconds, the eyes monitor the emerging translation (diamonds in green), presumably to control the produced output, while towards the end of the sentence, the gaze only seems to fixate the ST words (circles in blue) for which translations are currently typed. This typing behaviour exemplifies a high amount of production accuracy: the translation production in the successive TUs during this phase does not indicate hesitations, revision, or large corrections; on the contrary, it indicates that the translator is in tune with her predictions lowering the surprise during successive sensory input (reading). The translator seems to “settle into the right kind of attunement with the environment.” (Gallagher [57] (p. 160)).

A translator is in a steady translation state (S) if the translation production is at its optimum typing speed (which presumably depends on the translator’s typing skills). This implies that only a minimum amount of text is scanned, just enough that is required to keep a steady flow of translation production. A similar situation of translator–environment attunement with low variational free energy is shown in Figure 2. Previous research [16,26,62] suggests that experienced translators can—even without an initial orientation—become instantaneously attuned to a new translation situation and smoothly type out piece-by-piece translations whereby the eyes are only a few words ahead on the ST of the produced translations.

In contrast to these ‘low-surprise examples’, Figure 1 shows a slightly more effortful segment of translation production wherein a translator apparently starts out in a state of hesitation (H) or perhaps ‘overdetermination’ [60]. The course of the action suggests that the initial activity might continue pre-reflectively until the translator shift into an orientation phase (O) which settles the disattunement that is apparent during hesitation. It shows an instance in which the translator probably changed their mind and formulated a new translation solution.

## 7. Conclusions

This article connects the free energy principle (FEP, [27,28]) and active inference [29] with the monitor model [12] and relevance theory (RT, [24,25,46]) to explain human translation processes. Originally developed in theoretical neurosciences, FEP/active inference have been successfully applied in numerous fields, including artificial intelligence, sociology, economy, biology, philosophy, etc. [29] Cognitive translation studies (CTS), however, are lagging behind in this regard, although the principles, concepts, and standpoints seem compatible with human translation processes (see, e.g., [63]). FEP rests on the assumption that “any sentient creature must minimize the entropy of its sensory exchanges with the world”, where this entropy indicates the uncertainty or (expected or unexpected) surprise of the sensory input, and where “surprise can be expressed as a free energy function of sensations and (Bayesian) beliefs about their causes” (Friston et al. [50] (p. 2636)).

This paper suggests that reducing free energy in the translation process can be achieved by exploring the ST (i.e., expending reading effort) with the aim to activate appropriate mental resources that decrease divergencies between the internal and the external states. That is, an exploration may minimise the risks for successive translation production (see Equation (5)) if it minimises the discrepancy between the translator’s internal model (e.g., of the text) and their observations. An reduction in free energy can also be achieved as an effect of high translation production accuracy, i.e., through the exploitation of previously allocated resources, particularly if the ambiguity of the text to be translated is low.

In cases where an upcoming segment is expected to contain an elevated amount of ambiguities or difficulties, translators may first engage in an orientation phase, which enables them to allocate the appropriate mental resources. This would successively allow for increased production accuracy and a smooth translation flow (see, e.g., Figure 6). In case an unanticipated translation hurdle arises, translators may find themselves in a state of surprise or hesitation which may bring them (back) to an orientation phase (see, e.g., Figure 1). Translators can engage in successful and smooth translation production only when the average translation prediction error (i.e., the free energy) is sufficiently low. This seems to corroborate the opinion of Gutt [25], who arrived at similar conclusions, from the point of view of translator–audience interaction:

either the audience’s cognitive environment [i.e., their prior beliefs] needs to be adjusted so that it can process this information, or this information needs to be left aside in the higher-order communication act [i.e., in translation].

(Gutt [25] (p. 47))

The monitor model [12,42] stipulates the existence of (i) horizontal processes that trigger automatised translation routines; and (ii) vertical monitoring processes that activate reflective thought and search. Horizontal processes are assumed to be predominant in phases of fluent production, while monitoring processes are more likely to intervene in phases of hesitation or orientation. A similar distinction, albeit on a different theoretical background, has been suggested by [25,47], who proposed that translators may proceed in a stimulus mode (s-mode) accounting for “what was said” and/or an interpretive mode (i-mode), addressing “what was meant”. Translations would be produced in the s-mode if the source and the target audience shared large commonalities. S-mode translations may thus largely rely on horizontal, automatised routines, if the expected communication risks are assumed to be low and no further translation monitoring may be needed. The i-mode, in contrast, kicks in when bridging possible communication barriers, for instance, if the source and the target audience are members of different cognitive environments. Translators would be alert to activate higher-order monitoring processes that interfere when needed to ensure interpretive resemblance between the source and target texts. The monitor model is, thus, largely compatible with RT assumptions of the s-mode and i-mode; both models address similar phenomena from different angles (as can also be seen in [46]). The FEP/active inference, I suggest, provides a formal framework that has the potential to integrate both the monitor model and aspects of RT.

RT and the monitor model have emerged from different representationalist and non-representationalist assumptions. Luckily, the FEP/active inference is agnostic with respect to the nature of the processed representations. It does not (explicitly) address aspects of meaning construction, and can accommodate representationalist and non-representationalist views. FEP/active inference has been used to elicit different kinds of processes (e.g., neural and symbolic) across different domains and it has been instrumental to support representationalist [55] and non-representationalist [64] approaches in cognitive sciences. FEP/active inference is thus suited for modelling horizontal/priming/s-mode processes, as well as vertical/reflective/i-mode translation processes under a representationalist, ’classical’, and/or non-representationalist approaches to translational cognition.

Friston [27] points out that Markov blankets (sensation and action) provide a boundary that separates everything we are and do from everything ‘out there’ that may or may not exist. A posterior belief about the causes of my sensations may therefore be interpreted as representing or ‘standing in’ for a hypothesis that explains what I sense. This view is compatible with a representationalist approach. The symbols that are realised in the brain (or another computational device) stand in for something in the world; they are physical states that can be manipulated with truth-preserving rules. In this view, translation could be “understood as a rule-guided transformation of symbols from one code into the symbols of another code” (Martín de León [65] (p. 109)).

In contrast to a representationalist approach, an *enactivist* view on FEP (and active inference) rests on the assumption that action is biased to realise preferred outcomes, rather than higher-level representations. In this view, a “great bulk of world-directed, action-guiding cognition exhibits intentional directedness that is not contentful [...] It is possible that even sophisticated forms of human visual perceiving are not essentially contentful or representational” (Hutto and Myin [66] (p. 82)). Similarly, Bruineberg [64] (p. 11)) argues that:

what the agent is ‘modeling’ in a concrete situation is not so much the causal structure of the environment, but rather the relevant action possibilities that bring the agent closer to a self-generated optimum.

The intuition in active inference, he suggests, should not be how the brain uses sensory input to reconstruct the hidden state of affairs in the world, since “the space of possible ‘hypotheses’ is always already constrained and crooked in such a way as to make the animal tend to optimal conditions” (Bruineberg [64] (p. 11)). An agent will selectively sample the sensory inputs that it expects, since “only when I predict myself to be an agent acting in the world, and flourishing in my environment, does minimizing prediction-errors lead to a flourishing state” [64] (p. 10). The primary role of prediction error minimisation is not to infer and represent hidden causes in the world, but to bring about changes in the world that help the agent stay alive.

## Data Availability

The data can be freely downloaded as part of the CRITT TPR-DB: https://sites.google.com/site/centretranslationinnovation/tpr-db (accessed on 7 March 2023).
